# Expression of *sept3, sept5a and sept5b* in the Developing and Adult Nervous System of the Zebrafish (*Danio rerio*)

**DOI:** 10.3389/fnana.2017.00006

**Published:** 2017-02-17

**Authors:** Frederik Helmprobst, Christina Lillesaar, Christian Stigloher

**Affiliations:** ^1^Biocenter, Division of Electron Microscopy, University of WürzburgWürzburg, Germany; ^2^Biocenter, Department of Physiological Chemistry, University of WürzburgWürzburg, Germany

**Keywords:** septin, RNA *in situ* hybridization, neuronal development, retinal development, *sept3*, *sept5a*, *sept5b*

## Abstract

Septins are a highly conserved family of small GTPases that form cytoskeletal filaments. Their cellular functions, especially in the nervous system, still remain largely enigmatic, but there are accumulating lines of evidence that septins play important roles in neuronal physiology and pathology. In order to further dissect septin function in the nervous system a detailed temporal resolved analysis in the genetically well tractable model vertebrate zebrafish (*Danio rerio*) is crucially necessary. To close this knowledge gap we here provide a reference dataset describing the expression of selected septins (*sept3*, *sept5a* and *sept5b*) in the zebrafish central nervous system. Strikingly, proliferation zones are devoid of expression of all three septins investigated, suggesting that they have a role in post-mitotic neural cells. Our finding that three septins are mainly expressed in non-proliferative regions was further confirmed by double-stainings with a proliferative marker. Our RNA *in situ* hybridization (ISH) study, detecting *sept3*, *sept5a* and *sept5b* mRNAs, shows that all three septins are expressed in largely overlapping regions of the developing brain. However, the expression of *sept5a* is much more confined compared to *sept3* and *sept5b*. In contrast, the expression of all the three analyzed septins is largely similar in the adult brain.

## Introduction

Septins, a highly conserved family of small GTPases that form filaments, have various functions in normal physiology as well as pathology (Mostowy and Cossart, 2012; Dolat et al., 2014). For instance, septins are reported to play a role in cytokinesis, axon growth and exocytosis. Furthermore, they are involved in developmental processes, such as left-right-asymmetry regulation, as well as several pathologies, such as cancer, neurodegenerative diseases and psychiatric disorders (Dash et al., 2014; Dolat et al., 2014; Zhai et al., 2014; Marttinen et al., 2015). However, the cellular roles and functions of septins, in particular how they are involved in the physiology of the nervous system, still need to be fully elucidated.

Septins were first discovered in cell division screens in budding yeast (Hartwell et al., 1970) and named after their localization at the septum (Byers and Goetsch, 1976; Sanders and Field, 1994). Interestingly, septin homologs were identified later on in other eukaryotes like fungi, worms, flies, zebrafish and mammals (Cao et al., 2007; Pan et al., 2007; Nishihama et al., 2011). In humans, there are 13 septin genes, which can be divided according to sequence similarity into four homology groups: SEPT2 (SEPT1, SEPT2, SEPT4 and SEPT5), SEPT3/9 (SEPT3, SEPT9 and SEPT12), SEPT6 (SEPT6, SEPT8, SEPT10, SEPT11 and SEPT14), and SEPT7 (SEPT7; Kinoshita, 2003; Dolat et al., 2014). In the zebrafish (*Danio rerio*) genome, there are currently 17 septin genes annotated (Willis et al., 2016). The increased diversity of septins is likely due to an teleost specific genome duplication event (Postlethwait et al., 2004), so that there are for instance two homologs of SEPT5 (*sept5a* and *sept5b*) present in the zebrafish genome (Willis et al., 2016). In addition, genes like zebrafish *sept9a* and *sept9b* also have additionally multiple isoforms due to alternative splicing (Landsverk et al., 2010). The overall structure of septins is highly conserved. They share a phosphoinositide-binding polybasic domain, followed by the GTP-binding sites and the Septin Unique Element (SUE). A coiled-coil domain is found in septins belonging to the SEPT2, SEPT6 and SEPT7 groups. Furthermore, septins form heterofilaments consisting of two or more types of septins (Kinoshita, 2003; Mostowy and Cossart, 2012; Dolat et al., 2014).

In the mammalian central nervous system Septin3 and Septin5 have been attributed specific roles. Septin3 (Xue et al., 2004) and Septin5 (Kinoshita et al., 2000) are enriched at presynaptic terminals. Septin5 can inhibit exocytosis and interact with presynaptic proteins (Beites et al., 1999, 2005). Furthermore, Septin5 function has an effect on social behavior of mice (Suzuki et al., 2009; Harper et al., 2012). Septin5 is located in the human genome at position 22q11.2, a region that is the site of the most common micro-deletion syndrome in humans. This deletion is associated with multiple phenotypes including cardiac and palatal anomalies, intellectual disabilities and other disorders affecting the nervous system (Guna et al., 2015). Moreover, for Septin5 a fine-regulated expression at later developmental stages has been reported (Maldonado-Saldivia et al., 2000).

Despite these indications of the important roles of Septin3 and Septin5 in brain function and development, comparably little is known about the temporal expression of these genes during early vertebrate brain development. Previously, it was shown that zebrafish Septin5b is expressed in 1 day post-fertilization (dpf) embryos (Gomez et al., 2012) and Septin3 is generally expressed in neural tissues (Thisse and Thisse, 2004). However, a precise temporal description of septin expression in early vertebrate brain development is currently missing. Such information is necessary and opens up a high resolution view into the cellular role of septins in neurons.

The zebrafish is an established model organism well suited for studies of early vertebrate brain development (Wullimann et al., 1996; Mueller and Wullimann, 2015) and activity (Akerboom et al., 2012). Due to the accessible development and the large number of transparent offspring, zebrafish is a very fitting model for the systematic investigation of gene expression during development using RNA *in situ* hybridization (ISH; Thisse and Thisse, 2008). In addition to that, zebrafish is very suited for systematic high resolution and functional analysis to shed light on the cellular roles of septins in neurons. The zebrafish is a well-established neurogenetic model with a rich genetic toolbox (Wullimann et al., 1996; Lillesaar, 2011; Mueller and Wullimann, 2015; Willis et al., 2016). These are key advantages of this model, as septins seem to play important roles in synaptic development (Yang et al., 2010).

Here we present a precise description of the expression patterns of *septin3, septin5a* and *septin5b* at different developmental stages of zebrafish larvae as well as in adult brains. This study builds the basis for a thorough analysis of the role of these septins in the nervous system using the vertebrate model organism zebrafish (*Danio rerio*).

## Materials and Methods

### Zebrafish Culture and Nomenclature

Zebrafish (*Danio rerio*) larvae and adults (4–6 months) of the wild-type strain (AB) were kept on a day/night cycle of 14 h light and 10 h darkness. Larvae were kept at 28.5°C and staged as reported previously (Kimmel et al., 1995) and were raised in 30% Danieau’s solution (Westerfield, 2000). The pigmentation of zebrafish larvae was inhibited by 0.2 mM 1-phenyl-2-thiourea treatment (Karlsson et al., 2001). The brain structure nomenclature is conforming to reference atlases (Wullimann et al., 1996; Mueller and Wullimann, 2015). All experiments were performed according to the animal welfare regulations of the District Government of Lower Franconia.

### Phylogenetic Analyses

The human homologs of zebrafish Sept3 (NP_001019589.1), Sept5a (NP_956282.1), and Sept5b (NP_001003782.1) sequences (Willis et al., 2016) were aligned to Septin3 and Septin5 protein sequences from mouse (Sept3: NP_036019.2; Sept5: NP_998779.2) and human (Sept3 isoform B: NP_061979.3; Sept5 isoform 1: NP_002679.2), as well as to the two known septins from *C. elegans* (UNC-59: NP_493388.1; UNC-61 isoform a: NP_872156.2). Alignment was performed by the “One Click” Method using the web tool with GBlocks^1^ (RRID:SCR_010266, Dereeper et al., 2008) combining several algorithms (Castresana, 2000; Guindon and Gascuel, 2003; Edgar, 2004; Anisimova and Gascuel, 2006; Chevenet et al., 2006; Dereeper et al., 2010).

### RNA Extraction and Reverse Transcription

Zebrafish RNA was isolated from 1 to 4 dpf zebrafish larvae using the RNeasy Mini Kit (Qiagen Gmbh, Düsseldorf, Germany). The RevertAid First Strand cDNA Synthesis Kit (Thermo Scientific, Braunschweig, Germany) was used to generate cDNA.

### Molecular Cloning

Specific PCR primers for *sept3* (RefSeq: NM_001024418), *sept5a* (RefSeq: NM_199988), and the forward primer (fwd) for *sept5b* (RefSeq: NM_001003782) were generated.

The *sept5b* reverse primer (rev, CGGTCCTGCTGCTTCGGCTC) was designed according to previously published work (Gomez et al., 2012).

Primers were ordered from Sigma-Aldrich (Munich, Germany):

sept3_fwd (ATGTCAGAAATTGTGCCCCCTGAAGTGA),

sept3_rev (TCACAGGTTGCTTTCTTGTGTATC),

sept5a_fwd (ATGACGACCAACATCCGATACAAGAGCA),

sept5a_rev (TCACTGGTCTTTCTCGTGCATCTG), and

sept5b_fwd (ATGACGAGCAGCGCCAGGTACAAGAGCA).

Septin sequences were amplified from a cDNA mix of 1–4 dpf zebrafish larvae and cloned into the pJET-plasmid using the CloneJET PCR Cloning Kit (Thermo Scientific, Braunschweig, Germany) and verified by sequencing by GATC Biotech AG (Konstanz, Germany). To generate plasmids for RNA ISH probe generation, the septin sequences were cut out of the pJET-plasmids using the restriction enzymes XbaI and XhoI (Thermo Scientific, Braunschweig, Germany) and cloned into the pSC-A-amp/kan plasmid (Agilent Technologies, Santa Clara, CA, USA).

### RNA *In Situ* Hybridization

Digoxigenin-labeled sense and antisense probes were generated using T3 and T7 RNA Polymerases and the DIG RNA Labeling Kit (Roche, Mannheim, Germany). Whole mount RNA ISH was performed on zebrafish larvae and adult brains fixed with 4% PFA as previously published (Thisse et al., 1993; Thisse and Thisse, 2008). After RNA ISH adult brains were embedded in 3% agarose in PBS after stringency washes and 90 μm sections were cut using a TPI Vibratome 1000 Tissue Sectioning System (Technical Products International, Inc. St. Louis, MO, USA). Digoxigenin-labeled probes were detected with an anti-dig-AP-antibody (final dilution 1:5000, Roche, Mannheim, Germany) and revealed with NBT/BCIP (Roche, Mannheim, Germany) or SIGMAFAST Fast Red TR/Naphthol AS-MX Tablets (Sigma-Aldrich, Munich, Germany). Larvae were embedded in Epon (Westerfield, 2000) after RNA ISH and 8 μm sections were cut on a Leica EM UC7 (Leica Microsystems GmbH, Wetzlar, Germany) with a histology diamond knife (Dianova, GmbH, Hamburg, Germany). The Epon sections were counterstained with Hoechst 33342 (Sigma-Aldrich, Munich, Germany, 5 mg/μl stock solution) diluted 1:250.

### Immunocytochemistry and Microscopy

Immunocytochemistry was performed after RNA ISH on larvae or on adult brain sections following a previously described protocol (Yamamoto et al., 2011). Primary monoclonal anti-Tyrosine Hydroxylase (TH, clone LNC1, MERCK Millipore, Darmstadt, Germany) mouse antibody purified from PC12 cells was used to identify catecholaminergic neurons (Yamamoto et al., 2011). To identify proliferating cells a monoclonal anti-proliferating cell nuclear antigen (PCNA, Clone PC10, Dako, Glostrup, Denmark) mouse antibody was used (Mueller and Wullimann, 2015). As secondary antibody the donkey anti-mouse Alexa-488 IgG (H + L) antibody (Jackson ImmunoResearch Labratories, Suffolk, UK) was applied. Primary antibodies were used in a dilution of 1:100, the secondary antibody in a dilution of 1:500. Microscopy images were obtained with a Leitz Aristoplan or a Leica TCS SP confocal microscope (Leica Microsystems GmbH, Wetzlar, Germany).

## Results

### Zebrafish Septin3 and 5 Domain Structure and Conservation

**Figure 1:**
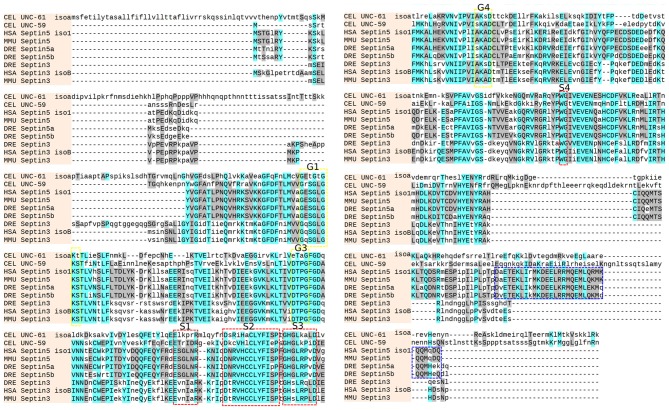
**Sequence alignment.** The sequences from *C. elegans* (CEL UNC-59 and CEL UNC-61 isoform a), mouse (MMU Septin3 and MMU Septin5), human (HSA Septin3 isoform B and HSA Septin5 isoform 1) and zebrafish (DRE Septin3, DRE Septin5a and DRE Septin5b) were aligned with the online tool (http://www.phylogeny.fr/). Similar residues are colored as the most conserved according to BLOSUM62 (cyan: max 3.0 and gray: low 0.5). The G1, G3 and G4 motifs of the GTPase domain (yellow, Kinoshita, 2003) as well as the conserved S1, S2, S3 and S4 motifs (red, Pan et al., 2007) and the coiled-coil domain of the Septin5 group (blue) are highlighted.

Zebrafish Sept3, Sept5a and Sept5b protein sequences were aligned and show high conservation with their mouse and human homologs (Figure 1). The conserved GTPase domains are present in the zebrafish sequences, specifically the G1 (GXXXXGKS), G3 (DXXG) and G4 (XKXD) motifs described previously (Kinoshita, 2003). Similarly, the S1 (EXXXXR), S2 (DXR(V/I)HXXX(Y/F)F(I/L)XP), S3 (GXXLXXXD), and S4 (WG) motifs are present (Pan et al., 2007; Figure 1). Additionally, the alignment shows that zebrafish Sept5a and Sept5b contain a structured coiled-coil domain at the C-terminus, whereas Sept3 is missing this structure, as reported previously (Kinoshita, 2003).

### Expression Patterns of *sept3*, *sept5a* and *sept5b* in Zebrafish Larvae during Development

**Figure 2:**
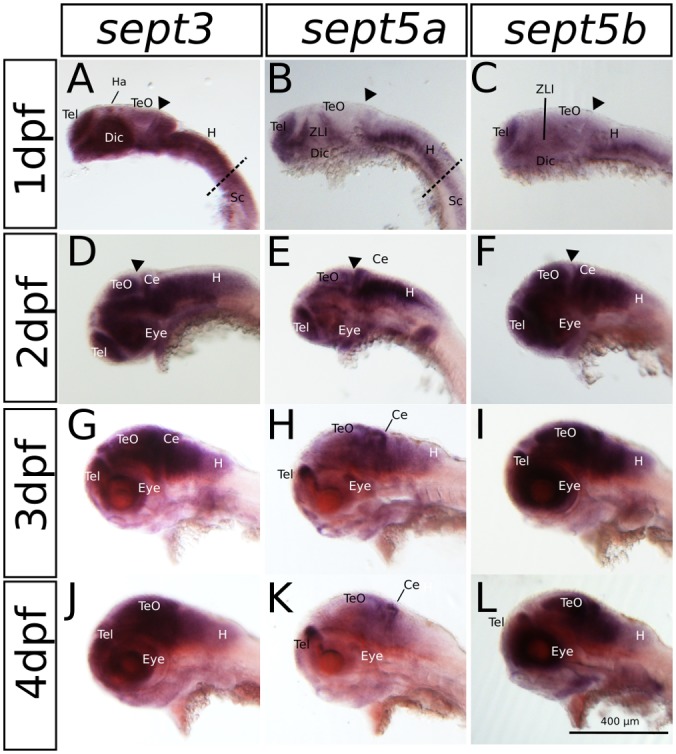
**The expression pattern of *sept3*, *sept5a* and *sept5b* in 1–4 dpf zebrafish larvae in a lateral view.** *Sept3*, *sept5a* and *sept5b* expression is shown by ISH, the blue color indicates the cells expressing the mRNA of each septin. The expression of *sept3* is shown in 1 dpf **(A)**, 2 dpf **(D)**, 3 dpf **(G)** and 4 dpf **(J)** zebrafish larvae. *Sept5a* expression is shown in 1 dpf **(B)**, 2 dpf **(E)**, 3 dpf **(H)** and 4 dpf **(K)** larvae. Zebrafish larvae 1–4 dpf stained with the *sept5b* probe are shown in **(C,F,I,L)**. The telencephalon (Tel), habenula (Ha), diencephalon (Dic), the zona limitans intrathalamica (ZLI), cerebellum (Ce), tectum (TeO), eye, the spinal cord (Sc) as well as the hindbrain (H) are labeled. The border between hindbrain and spinal cord is marked by a dashed line. The midbrain-hindbrain boundary (MHB) is labeled (black arrowhead).

**Figure 3:**
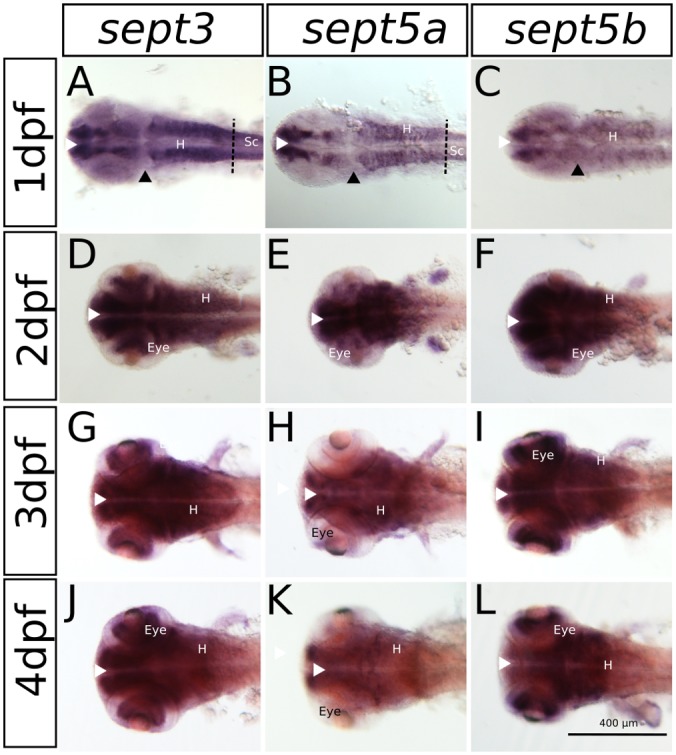
**The expression pattern of *sept3*, *sept5a* and *sept5b* in 1–4 dpf zebrafish larvae in a dorsal view.** *Sept3*, *sept5a* and *sept5b* expression is shown by ISH, the blue color indicates the cells expressing the mRNA of each septin. The expression of *sept3* is shown in 1 dpf **(A)**, 2 dpf **(D)**, 3 dpf **(G)** and 4 dpf **(J)** zebrafish larvae. *Sept5a* expression is shown in 1 dpf **(B)**, 2 dpf **(E)**, 3 dpf **(H)** and 4 dpf **(K)** larvae. Zebrafish larvae 1–4 dpf stained with the *sept5b* probe are shown in **(C,F,I,L)**. The eye, spinal cord (Sc) and hindbrain (H) are marked. The border between hindbrain and spinal cord is marked by a dashed line. Ventricular zones (white arrowhead) and the MHB (black arrowhead) are labeled.

**Figure 4:**
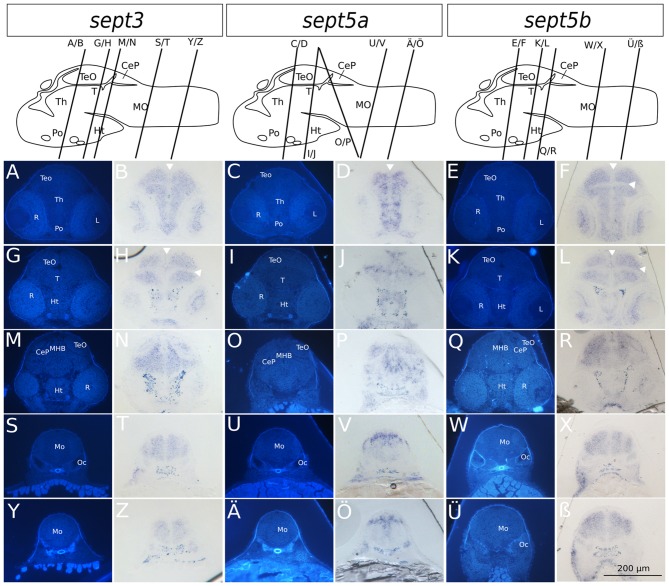
**Transverse sections through 2 dpf zebrafish larvae stained with ISH probes against *sept3*, *sept5a* and *sept5b*.** 2 dpf zebrafish larvae were stained for *sept3* **(A,B,G,H,M,N,S,T,Y,Z)**, *sept5a* **(C,D,I,J,O,P,U,V,Ä,Ö)** and *sept5b* **(E,F,K,L,Q,R,W,X,Ü,**ß**)**, embedded in epon, and cut in 8 μm thick section. The schemes (modified from Mueller and Wullimann, 2015) are showing the sagittal views of a 2 dpf zebrafish brain with indicated section planes. For better orientation the ISH sections were counterstained with Hoechst **(A,G,M,S,Y,C,I,O,U,Ä,E,K,Q,W,Ü)**. Septin expression is indicated by the blue color **(B,H,N,T,Z,D,J,P,V,Ö,F,L,R,X,ß)**. The retina (R), tectum opticum (TeO), tegmentum (T), preoptic region (Po), thalamus (Th), hypothalamus (Ht), medulla oblongata (Mo), cerebella plate (CeP), MHB and otic capsule (Oc), are marked. The ventricles are indicated with white arrowheads. Schemes for better orientation are modified from Mueller and Wullimann (2015) 2 dpf schemes.

**Figure 5:**
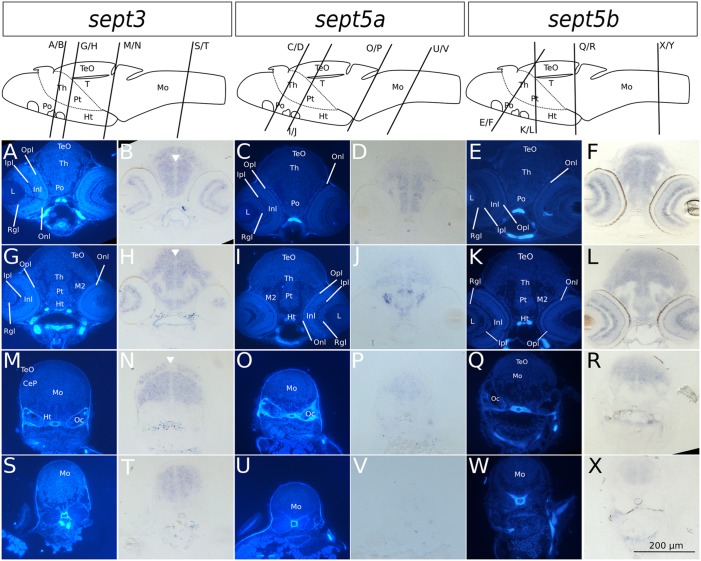
**Transverse sections through 4 dpf zebrafish larvae stained with ISH probes against *sept3*, *sept5a* and *sept5b*.** 2 dpf zebrafish larvae were stained for *sept3* **(A,B,G,H,M,N,S,T)**, *sept5a* **(C,D,I,J,O,P,U,V)** and *sept5b* **(E,F,K,L,Q,R,W,X)**, embedded in epon, and cut in 8 μm thick section. For better orientation, the ISH sections were counterstained with Hoechst **(A,G,M,S,C,I,O,U,E,K,Q,W)**. Septin expression is indicated by the blue color **(B,H,N,T,D,J,P,V,F,L,R,X)**. The tectum (TeO), preoptic region (Po), thalamus (Th), hypothalamus (Ht), cerebellum (Ce), the medulla oblongata (Mo) and the otic capsules (Oc), are marked. Additionally the migrated posterior tubercular area (M2) and the posterior tuberculum (Pt) are annotated. In the eye, the retinal ganglion cell layer (Rgl), the inner and outer nuclear layer (Inl and Onl), as well as the inner and outer plexiform layer (Ipl and Opl) are shown. The ventricles are indicated with white arrowheads. Schemes for better orientation with indicated section planes are modified from Mueller and Wullimann (2015) 5 dpf schemes, as these are structurally very close to the here shown 4 dpf sections.

The expression patterns of *sept3, sept5a* and sept*5b*, as analyzed by whole mount RNA ISH, in the head region of 1–4 dpf zebrafish larvae are shown in lateral views in Figure 2 and dorsal views in Figure 3. At 1 dpf, *sept3* is expressed in the telencephalon (Tel), the diencephalon (Dic), the hindbrain (H), and the spinal cord (Sc; Figures 2A, 3A). Furthermore, *sept3* expression is detectable in the habenula (Ha). The tectum opticum (TeO), the mid-hindbrain boundary (MHB), the retina (R) and the periventricular regions are devoid of notable expression. From 2 dpf to 4 dpf, *sept3* expression can be found additionally in TeO, cerebellum (Ce), and the retina (Figures 2D,G,J, 3D,G,J). In the ventricular zones (Figures 4, 5) no *sept3* expression can be observed during development up to 4 dpf.

At 1 dpf, *sept5a* is expressed in the telencephalon and the diencephalon (Figures 2B, 3B). In the diencephalon the zona limitans intrathalamica (ZLI) remains unstained. *Sept5a* is further expressed in the rhombomeres of the hindbrain and in the Sc. At 2 dpf *sept5a* is expressed in the TeO (Figures 2E, 3E). In the retina no *sept5a* expression is detectable after 2 dpf. From 2 dpf to 4 dpf the expression of *sept5a* decreases in most brain regions, but is detectable in the telencephalon and the dorsal part of the Ce (Figures 2H,K, 3H,K). No *sept5a* expression can be detected in the ventricular zones (Figures 4, 5).

At 1 dpf, *sept5b* is expressed in the telencephalon, diencephalon, and hindbrain (Figures 2C, 3C). In the ZLI and TeO *sept5b* is not expressed. From 2 dpf to 4 dpf *sept5b* is additionally detectable in the Ce, the TeO and the retina (Figures 2F,I,L, 3F,I,L). Similar to *sept5a*, *sept5b* expression is absent from the ventricular zones (Figures 4, 5).

Summing up, during early development the selected septins are expressed broadly in the brain but proliferative zones, such as ventricular zones in general, and the ZLI and MHB (Figure 4) in particular, seem to be omitted.

### *Sept3*, *sept5a* and *sept5b* Expression in Brain Sections of 2 and 4 dpf Zebrafish Larvae

To analyze the developmental expression of the septins in greater spatial detail, cross sections through the head of 2 and 4 dpf larvae were prepared and counterstained with Hoechst (Figures 4, 5). At 2 dpf *sept3* is expressed broadly outside the ventricular zones in the TeO (Figures 4A,B,G,H,M,N), the preoptic region (Po; Figures 4A,B), the thalamus (Th; Figures 4A,B), the hypothalamus (Ht; Figures 4G,H,M,N), and the medulla oblongata (Mo; Figures 4S,T,Y,Z), as well as in the developing retina (R; Figures 4A,B,G,H,M,N). While *sept5b* (Figures 4E,F,K,L,Q,R,W,X,Ü,ß) shows a very similar expression at 2 dpf, *sept5a* is expressed differently (Figures 4C,D,I,J,O,P,U,V,Ä,Ö). *Sept5a* is expressed in stripe-like patterns with alternating high and low expressions along the dorso-ventral axis in TeO (Figures 4C,D,I,J,O,P), T (Figures 4I,J), Po (Figures 4C,D), Th (Figures 4C,D), Ht (Figures 4I,J), and Mo (Figures 4U,V,Ä,Ö). The expression of *sept5a* is more restricted to regions near the ventricles, but there is only patchy expression in the ventricular zone itself. Furthermore, the retina shows faint *sept5a* expression.

In 4 dpf zebrafish larvae, *sept3* is expressed in the retinal ganglion cell layer (Rgl) and the inner nuclear layer (Inl), (Figures 5A,B,G,H). In the brain proper, *sept3* is expressed in the TeO (Figures 5A,B,G,H,M,N), Po (Figures 5A,B), Th (Figures 5A,B,G,H), Ht (Figures 5G,H), and Mo (Figures 5M,N,S,T), as well as strongly in the migrated posterior tubercular area (M2; Figures 5G,H). No *sept3* expression can be found in the ventricular zones, or in the posterior tuberculum (Pt; Figures 5G,H). *Sept5a* is strongly expressed in the Po (Figures 5C,D), M2, Ht and dorsally in the Pt (Figures 5I,J). A weaker expression is detectable in the TeO, Th (Figures 5C,D,I,J), and Mo (Figures 5O,P,U,V). No *sept5a* expression was detected in the eye or the ventricles. The expression of *sept5b* resembles the expression pattern of *sept3*. *Sept5b* is expressed in the Rgl and Inl of the retina (Figures 5E,F,K,L). In the brain proper, there is strong expression of *sept5b* in the TeO (Figures 5E,F,K,L), Po (Figures 5E,F), Th (Figures 5E,F,K,L), M2, Ht, Pt (Figures 5K,L), and Mo (Figures 5Q,R,W,X). Interestingly, the expression of both genes is high in the dorsal and low in the ventral regions of the Mo (Figures 5M,N,S,T,Q,R,W,X).

**Figure 6:**
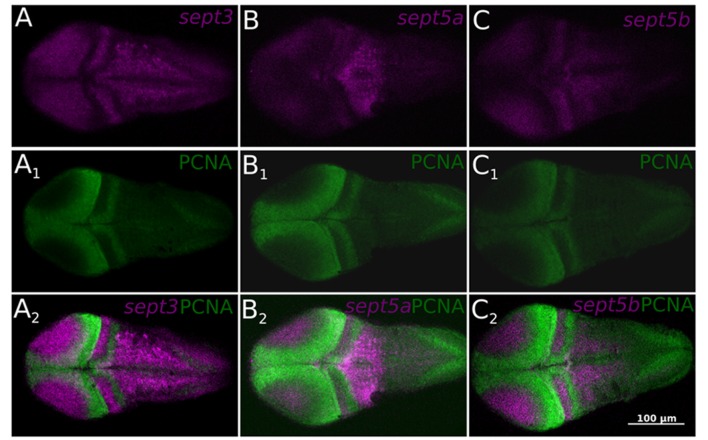
**Single confocal section trough 2 dpf zebrafish larvae stained with ISH probes against *sept3* (A)**, *sept5a* **(B)**, and *sept5b* **(C)** from a dorsal view. The larvae were additionally stained with an antibody against Proliferating Cell Nuclear Antigen (PCNA; **A1,B1,C1**). All three septins (magenta) are mostly not expressed in proliferating cells (green, **A2,B2,C2**).

To clarify, if the three septin genes are expressed in proliferation zones, an immunohistochemistry staining was performed on ISH stained 2 dpf zebrafish larvae with an antibody against PCNA (Figure 6), which confirms, that the three septin genes are mostly not expressed in proliferation zones.

To summarize, this more fine resolved analysis on thin sections and co-stainings with a well-established proliferative marker strongly supports the aforementioned notion that *sept3*, *sept5a* and *sept5b* expression is absent from proliferative zones.

### *Sept3*, *sept5a* and *sept5b* Expression in Adult Brains

**Figure 7:**
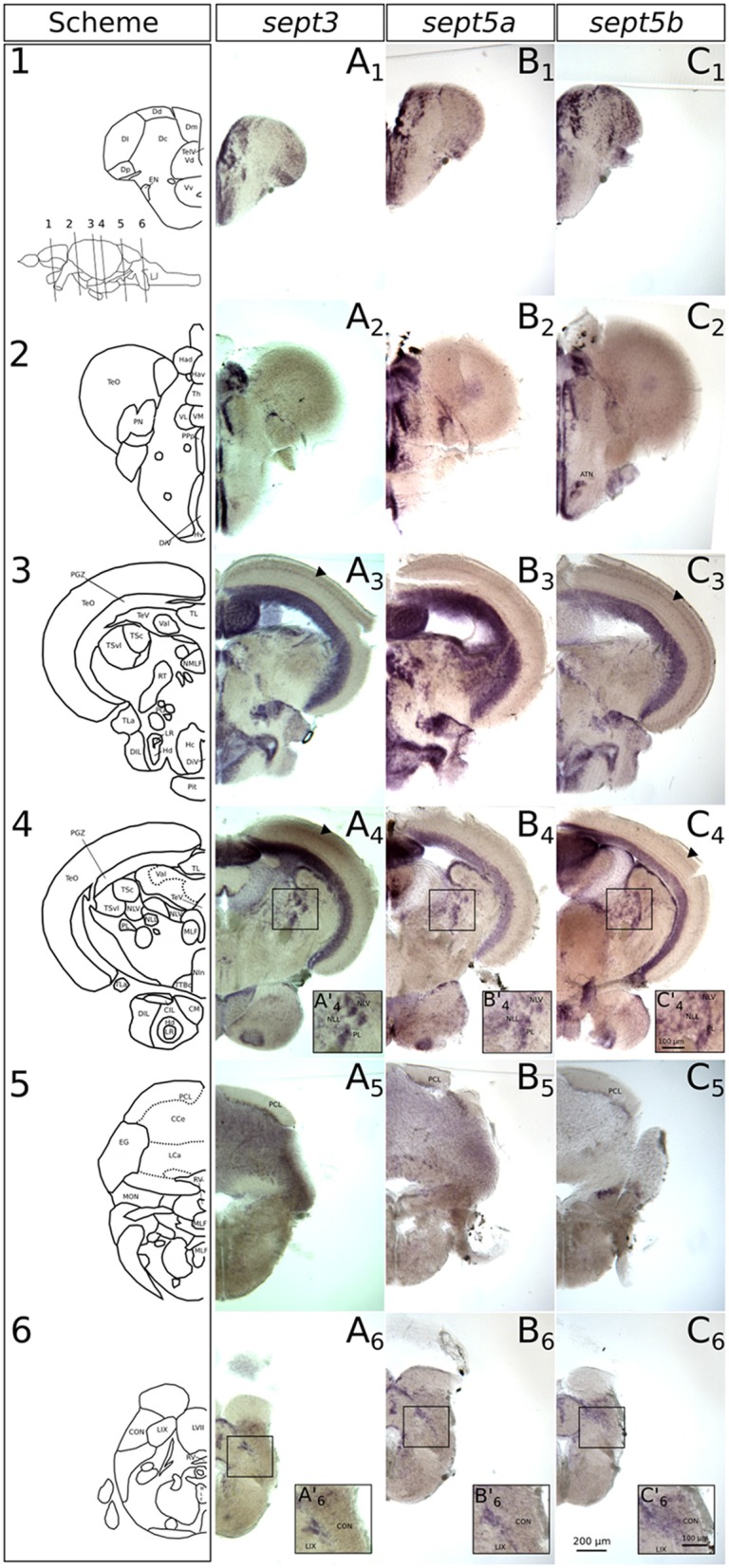
**Expression of *sept3*, *sept5a* and *sept5b* in adult zebrafish brains.** The expression pattern of *sept3* **(A1–A6)**, *sept5a* **(B1–B6)** and *sept5b* **(C1–C6)** in adult brain sections are shown. The schemes **(1–6)** show corresponding sections from the zebrafish brain atlas (modified from Wullimann et al., 1996). Abbreviations can be found in Table 1. The anterior tuberal nucleus (ATN) is indicated in **(C_2_)**. The *sept3* and *sept5b* positive layer in the TeO is labeld with black arrowheads. The inlays in **(A’_4_,B’_4_,C’_4_,A’_6_,B’_6_,C’_6_)** are showing magnified views of the boxed regions.

**Figure 8:**
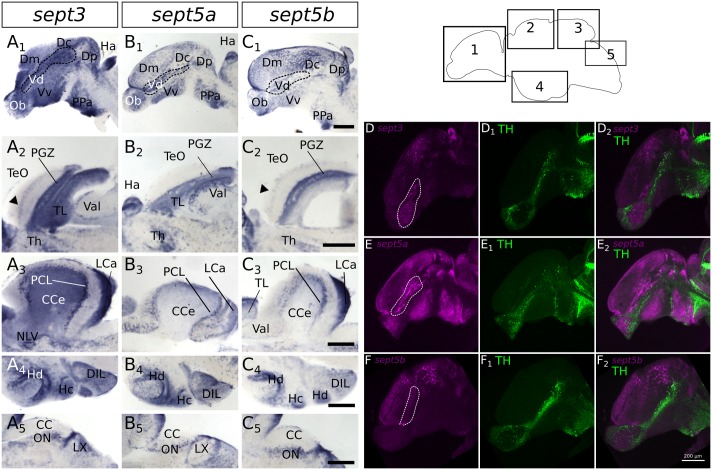
**Expression of *sept3*, *sept5a* and *sept5b* in substructures of the adult zebrafish brain.** The expression pattern of *sept3* **(A1–A5)**, *sept5a* **(B1–B5)**, and *sept5b* **(C1–C5)** is shown in sagittal sections of adult brains. The regions of the brain are indicated in the scheme **(1–5)**. In the telencephalon **(1)** *sept3*, *sept5a* and *sept5b* are expressed in dorsal telencephalic areas (Dm, Dc and Dp) and ventral telencephalic areas (Vd and Vv). Septins are expressed in a stream in the Vd region (outlined by dotted line). Additionally, *sept3*, *sept5a* and *sept5b* is expressed in the Ob. The *sept3* and *sept5b* positive layer in the TeO is labeld with black arrowheads **(A2,C2)**. In the cerebellum **(3,5)** *sept3*, *sept5a* and *sept5b* is expressed in the LCa, and in the region of the purkinje cell layer (PCL). *Sept3* is additionally expressed in the CCe. Furthermore, hypothalamic sagittal section are shown **(4)**. Abbreviations can be found in Table 1. To identify dopaminergic cells, ISH stained brains (magenta) against *sept3* **(D)**, *sept5a* **(E)**, and *sept5b* **(F)** were counterstained with an antibody against tyrosine hydroxylase (TH, green in **D1,E1,F1**) and analyzed with a confocal microscope and merged **(D2,E2,F2)**. Pictures in **(D–F)** are maximum projections of confocal slices through adult sagittal brain sections. Scale bars 200 μm.

In order to compare septin expression at early stages with the mature nervous system, we analyzed the expression patterns of *sept3*, *sept5a* and *sept5b* in adult brains with ISH (Figures 7, 8). *Sept3, sept5a and sept5b* are expressed in pallial and subpallial regions of the telencephalon (Figures 7A1,B1,C1, 8A1,B1,C1). In the pallium, transcripts are found in the medial zone of dorsal telencephalic area (Dm) near the ventricle and in the posterior zone of dorsal telencephalic area (Dp; Figures 7A1,B1,C1). Transcripts are also detectable in the lateral zone of dorsal telencephalic area (Dl) and the dorsal zone of dorsal telencephalic area (Dd), but exhibit a more scattered distribution (Figures 7A1,B1,C1). Additionally, septin expression can be found in the central zone of dorsal telencephalic area (Dc; Figures 8A1,B1,C1). In the subpallium, expression is present in the entopeduncular nucleus (EN), dorsal nucleus of the ventral telencephalic area (Vd) and the ventral nucleus of the ventral telencephalic area (Vv; Figures 7A1,B1,C1). In a sagittal view of the telencephalon, expression of *sept3, sept5a* and *sept5b* can be found in a stripe in Vd region, as well as in the olfactory bulb (Ob; Figures 8A1,B1,C1). To determine, whether the stripe of cells in the telencephalon are mature dopaminergic neurons (Yamamoto et al., 2011) or part of the differentiating cells in the telencephalon (Grandel et al., 2006), immunohistochemistry staining with an antibody against TH was performed after ISH on adult brain sections (Figures 8D–F). This experiment shows, that *sept3*, *sept5a* and *sept5b*, seem to be not expressed directly within the stream of dopaminergic neurons in the telencephalon but in close proximity.

**Table 1:** **List of abbreviations**.

Abbreviation	Complete terminology
ATN	Anterior tuberal nucleus
CC	Crista cerebellaris
CCe	Corpus cerebelli
Ce	Cerebellum
CeP	Cerebellar plate
CIL	Central nucleus of the inferior lobe
CM	Corpus mamillare
CON	Caudal octavolateralis nucleus
D	Dorsal telencephalic area
Dc	Central zone of D
Dd	Dorsal zone of D
DI	Lateral zone of D
Dic	Diencephalon
DIL	Diffuse nucleus of the inferior lobe
DiV	Diencephalic ventricle
Dm	Medial zone of D
Dp	Posterior zone of D
dpf	Days post-fertilization
EG	Eminentia granularis
EN	Entopeduncular nucleus
Ep	Epiphysis
H	Hindbrain
Had	Dorsal habenular nucleus
Hav	Ventral habenular nucleus
Ha	Habenula
Hc	Caudal zone of periventricular hypothalamus
Hd	Dorsal zone of periventricular hypothalamus
Ht	Hypothalamus
Hv	Ventral zone of periventricular hypothalamus
IMRF	Intermediate reticular formation
Inl	Inner nuclear layer
Ipl	Inner plexiform layer
ISH	*in situ* hybridization
LCa	Lobus caudalis cerebelli
LIX	Lobus glossopharyngeus
LR	Lateral recess of diencephalic ventricle
LVII	Lobus facialis
LX	Vagal lobe
M2	Migrated posterior tubercular area
MHB	Midbrain-hindbrain boundary
MLF	Medial longitudinal fascicle
Mo	Medulla oblongata
MON	Medial octavolateralis nucleus
NLL	Nucleus of the lateral lemniscus
Nln	Nucleus interpeduncularis
NLV	Nucleus lateralis valvulae
NMLF	Nucleus of MLF
Oc	Otic vesicle
Onl	Outer nuclear layer
Opl	Outer plexiform layer
PCL	Purkinje cell layer
PG	Preglomerular nuclei
PGZ	Periventricular gray zone of optic tectum
Pit	Pituitary
PL	Perilemniscal nucleus
Po	Preoptic region
PN	Pretectal nuclei
PPa	Parvocellular preoptic nucleus, anterior part
PPp	Parvocellular preoptic nucleus, posterior part
Pt	Posterior tuberculum
ON	Octavolateralis nucleus
R	Retina
Rgl	Retinal ganglion cell layer
RT	rostral tegmental nucleus
RV	Rhombencephalic ventricle
Sc	Spinal cord
T	Tegmentum
Tel	Telencephalon
TelV	Telencephalic ventricle
TeO	Tectum opticum
TeV	Tectal ventricle
Th	Thalamus
TL	Torus longitudinalis
TLa	Torus lateralis
TSc	Central nucleus of torus semicircularis
TSvl	Ventrolateral nucleus of torus semicircularis
V	Ventral telencephalic area
Val	Lateral division of valvula cerebelli
Vd	Dorsal nucleus of V
VL	Ventrolateral thalamic nucleus
VM	Ventromedial thalamic nucleus
Vv	Ventral nucleus of V
ZLI	Zona limitans intrathalamica

In the diencephalon (Figures 7A2–4,B2–4,C2–4), all three septins are expressed in nuclei of the habenula (Ha), such as dorsal habenular nucleus (Had) and ventral habenular nucleus (Hav). Furthermore, *sept3*, *sept5a* and *sept5b* are expressed in the pretectal nuclei (PN; Figures 7A2,B2,C2). Additionally, septin expression can be found in thalamic structures like the ventrolateral thalamic nucleus (VL; Figures 7A2,B2,C2), the ventromedial thalamic nucleus (VM; Figures 7A2,B2,C2), anterior and posterior part of the parvocellular preoptic nucleus (PPa; Figures 8A1,B1,C1) and (PPp; Figures 7A2,B2,C2), and the nucleus of the medial longitudinal fascicle (NMLF; Figures 7A3,B3,C3). Septins are also expressed in the preglomerular nuclei (PG; Figures 7A3,B3,C3) and in torus lateralis (TLa; Figures 7A3–4,B3–4,C3–4). In the Ht, septin expression can be found in the dorsal zone of the periventricular hypothalamus (Hd; Figures 7A3–4,B3–4,C3–4, 8A4,B4,C4), caudal zone of the periventricular hypothalamus (Hc; Figures 7A3,B3,C3, 8A4,B4,C4), and ventral zone of the periventricular hypothalamus (Hv; Figures 7A2,B2,C2). Furthermore, the septins are expressed in diffuse nucleus of the inferior lobe (DIL; Figures 7A3–4,B3–4,C3–4, 8A4,B4,C4) and the corpus mamillare (CM; Figures 7A4,B4,C4). Additionally *sept5b* expression can be found in the anterior tuberal nucleus (ATN; Figure 7C3).

In the mesencephalon (Figures 7A2–4,B2–4,C2–4), expression of all three septins is found in the periventricular gray zone of optic tectum (PGZ; Figures 7A3–4,B3–4,C3–4, 8A2,B2,C2). Further, *sept3* and *sept5b* show clearly two additional fine layers of expression in the TeO (Figures 7A3–4,C3–4, 8A2,B2,C2). Transcripts are also detectable in the central nucleus of torus semicircularis (TSc; Figures 7A3–4,B3–4,C3–4), torus longitudinalis (TL; Figures 7A3–4,B3–4,C3–4, 8A2,B2,C3), rostral tegmental nucleus (RT; Figures 7A3,B3,C3), nucleus of the lateral lemniscus (NLL) and perilemniscal nucleus (PL; Figures 7A4,B4,C4). Interestingly, *sept5b* is differentially expressed along the anterior-posterior axis of the TL with low expression anteriorly (Figure 7C3) and higher expression posteriorly (Figure 7C4). Additionally, *sept5a* is strongly expressed in the ventrolateral nucleus of torus semicircularis (TSvl; Figure 7B3). In the nucleus interpeduncularis (Nln; Figures 7A4,B4,C4) *sept3* is weakly expressed, whereas *sept5a* and *sept5b* are not detectable in this region.

Furthermore, *sept3*, *sept5a*, and *sept5b* are expressed in parts of the rhombencephalon (Figures 7A3–6,B3–6,C3–6, 8A3,A5,B3,B5,C3,C5). Expression is found in the lateral and medial part of the lateral division of valvula cerebelli (Val; Figures 7A3–4,B3–4,C3–4, 8A2,B2,C3) and the nucleus lateralis valvulae (NLV; Figures 7A4,B4,C4). Additionally, *sept3*, *sept5a* and *sept5b* are expressed in the lobus caudalis cerebelli (LCa; Figures 8A3,B3,C3), whereas only *sept3* is detectable in the corpus cerebelli (CCe; Figures 7A5,B5,C5, 8A3,B3,C3) and the eminentia granularis (EG; Figures 7A5,B5,C5). *Sept3*, *sept5a* and *sept5b* are expressed in the Purkinje cell layer (PCL; Figures 7A5,B5,C5, 8A3,B3,C3). Moreover, there is expression of *sept3, sept5a* and *sept5b* at the superficial layer of the lobus facialis (LVII; Figures 7A6,B6,C6), centrally in LVII and near to the ventricle where cell bodies are enriched and not in projection zones (Yáñez et al., 2017). Furthermore, there is expression of all three septins in a domain of the intermediate reticular formation (IMRF) adjacent to the MLF (Figures 7A6,B6,C6). Finally, the septins are expressed at the border between the caudal octavolateralis nucleus (CON) and the lobus glossopharyngeus (LIX; Figures 7A6,B6,C6). The sagittal sections (Figures 8A5,B5,C5) show, that *sept3*, *sept5a* and *sept5b* expression is detectable in the dorsal part of the octavolateralis nuclei (ON). More specifically, *sept5b* is expressed in the medial octavolateralis nucleus (MON; Figure 7C5).

Overall, and in contrast to early expression patterns of these three septin genes, where *sept5a* stood out, the adult brain shows an even more unified expression. The expression is summarized in Table 2.

**Table 2:** **Summary of *sept3*, *sept5a* and *sept5b* expression**.

Anatomic structure	*Sept3*	*Sept5a*	*Sept5b*
Dm	+	+	+
Dp	+	+	+
Dl	+	+	+
Dd	+	+	+
Dc	+	+	+
EN	+	+	+
Vd	+	+	+
Vv	+	+	+
Ob	+	+	+
Hb	+	+	+
Hav	++	++	++
Had	++	++	++
VL	+	+	+
VM	+	+	+
NMLF	+	+	+
PG	+	+	+
TLa	+	+	+
Hd	+	+	+
Hc	+	+	+
Hv	+	+	+
PPp	++	++	+
PPa	+	+	+
CM	+	+	+
DIL	+	+	+
ATN	?	?	+
PGZ	+	+	+
TSc	+	+	+
PN	+	+	+
TL	++	++	+
RT	+	+	+
NLL	+	+	+
PL	+	+	+
TSvl	−	++	−
NIn	+	−	−
Val	+	+	+
NLV	+	+	+
LCa	+	+	+
CCe	+	−	−
EG	+	−	−
PCL	+	+	+
LVII	+	+	+
IMRF	+	+	+
ON	+	+	+

*The level of Expression is indicated by no expression (−), expressed (+) and highly expressed (++). Unknown expression is indicated (?)*.

## Discussion

Evidence is accumulating that septins play important roles in neuronal function (Mostowy and Cossart, 2012; Dolat et al., 2014; Marttinen et al., 2015). In order to dissect the neuronal roles of septins, a reference dataset describing the expression of selected neuronally-expressed septins in a tractable vertebrate model like zebrafish is necessary. This work fills this knowledge gap and shows a detailed description of the expression patterns of zebrafish *sept3*, *sept5a* and *sept5b* during stages of early brain development as well as in the adult brain now providing a framework for the functional analysis of the roles of these septins in the nervous system of a tractable vertebrate model organism.

### Characteristic Differences in Early *Septin* Expression Patterns

While *sept3* and *sept5b* are similarly expressed during early development, a unifying principle of their expression patterns is that proliferative zones, in particular ventricular zones and the organizers at the ZLI and the MHB, stay free of *sept3* and *sept5b* expression. The expression of these two genes in the developing brain is largely overlapping with zones where the expression of the Hu protein has been described (Mueller and Wullimann, 2015), which hints to a role of *sept3* and *sept5b* in neuronal differentiation. While *sept5a* is mostly not expressed in the ventricular zones it seems to be expressed in a more specific pattern during early development. The expression pattern of *sept5a* in 2 dpf zebrafish larvae is more restricted and is downregulated in large parts of the brain during development to 4 dpf larvae, where it is strongly expressed in cells of the M2 area. Furthermore, in contrast to *sept3* and *sept5b*, no *sept5a* expression is detectable in the retina at 4 dpf. The observed expression of *sept5b* in the zebrafish retina is in accordance with immunohistochemistry studies of SEPT5 in human eye tissues (Pache et al., 2005; Xin et al., 2007), which suggests an important conserved role of Sept5 in retinal development (Xin et al., 2007). The *sept5b* expression differs from the expression of *sept3* in the retina. While both septins are expressed in the retinal ganglion layer, *sept3* is only expressed in the Inl in the region of the amacrine cells. Additional *sept5b* expressing cells can be found near to the Opl, where horizontal cells can be found (Gramage et al., 2014). In contrast to the defined differences during early development the expression of the three studied *septin* genes is much more similar in adult zebrafish brains. All three septin genes are expressed in regions with high proliferation activity in the adult brain (Grandel et al., 2006), but are not expressed in the proliferating zones themselves (Figure 6), which indicates a more specific role in neuronal differentiation processes, as it has been shown for Sept5 for rodent synaptogenesis (Yang et al., 2010).

Interestingly, the *sept5a* expressing cell clusters, especially in the thalamus of the zebrafish larvae (Figures 4, 5), resemble the distribution of GABAergic neurons (Mueller and Wullimann, 2015). Furthermore, this expression pattern is very similar to the expression of the metabotropic glutamate receptor (*mglur1a*). Additionally, *sept5a* and *mglur1a* are enriched in cells of the cerebellum in a similar pattern (Haug et al., 2013), hinting to a specific role of *sept5a* in a special subset of neurons.

### Comparison of Zebrafish and Mammalian *sept3, sept5a and sept5b* Expression

The human ortholog of Sept5 (HCDCREL-1) is expressed in neuronal tissues (Yagi et al., 1998). Furthermore, developing mouse embryos show strong neuronal and retinal expression of CDCrel-1 (Sept5; Maldonado-Saldivia et al., 2000). This expression seems to be more similar to the widely expressed *sept5b* in zebrafish than to the more restricted *sept5a* expression. The different expression patterns of the two *sept5* genes in zebrafish might be explained by subfunctionalization of the paralogs *sept5a* and *sept5b* after a specific genome duplication event in teleost fish (Postlethwait et al., 2004), where the paralogous genes were then free to diverge in their expression patterns. Interestingly differential expression of *Sept5* isoforms is described in different regions of mouse brains (Asada et al., 2010). In adult mouse, *Sept3* and *Sept5* are broadly expressed in the whole brain^2^, showing a particular intense expression in the cerebellum in the region of the PCL. Similar findings were described in immunoreactivity studies for *Sept3* in rat brain (Xue et al., 2004) and are in line with the observed specific expression at the PCL in adult zebrafish brains for *sept3*, *sept5a* and *sept5b*.

### The Cellular Role in Neurons of *sept3, sept5a* and *sept5b*

Notably, the orthologous proteins Sept3 and Sept5 are located at presynaptic terminals in rodent brains, indicating a key role for synaptic function (Kinoshita et al., 2000; Xue et al., 2004; Yang et al., 2010; Tsang et al., 2011). Due to the highly specific Septin5 expression in the brain and, in particular, its localization at nerve terminals (Kinoshita et al., 2000; Xue et al., 2004; Yang et al., 2010; Tsang et al., 2011) the involvement of Sept5 in exocytosis (Beites et al., 1999, 2005), as well as the interaction of Sept5 with PARK2 (Zhang et al., 2000) has linked this particular Septin to diseases of the nervous system like, schizophrenia, epilepsy and degenerative diseases like Parkinson’s and Alzheimer’s (Dolat et al., 2014; Marttinen et al., 2015). In the same line, the overexpression of Sept5 leads to a degeneration of dopaminergic neurons (Dong et al., 2003), which provides further indications to important roles of septins in brain function and development, but the specific cellular roles are not yet fully elucidated. Interestingly, in *C. elegans*, where only the two septins *unc-59* and *unc-61* are described in the genome, both septins have been shown to be involved in the development of the nervous system. Mutants of these genes show several behavioral defects like uncoordinated movement and abnormal egg laying (Nguyen et al., 2000; Finger et al., 2003). Moreover, a mouse knock out of *Sept3* and *Sept5* shows no obvious neuronal defects during development (Tsang et al., 2008), but further detailed behavioral studies show, that *Sept5* has an influence on mouse affective, cognitive and social behavior (Suzuki et al., 2009; Harper et al., 2012). With our expression study of *sept3*, *sept5a* and *sept5b* in the zebrafish brain, we provide now a detailed analysis for further investigations into the functions of septins in vertebrates from the cellular level to behavior.

## Author Contributions

All authors had full access to all the data in the study and take responsibility for the integrity of the data and the accuracy of the data analysis. FH, CL and CS: study concept and design; analysis and interpretation of data; drafting of the manuscript; critical revision of the manuscript for important intellectual content; FH and CL: acquisition of data; CS: study supervision.

## Funding

This work was supported by Universitätsbund Würzburg (AZ14-48). CL is funded by the Program Chancengleichheit für Frauen in Forschung und Lehre from the Bayerische Gleichstellungsförderung (BGF) and University of Würzburg. This publication was funded by the German Research Foundation (DFG) and the University of Würzburg in the funding programme Open Access Publishing.

## Conflict of Interest Statement

The authors declare that the research was conducted in the absence of any commercial or financial relationships that could be construed as a potential conflict of interest.

## Footnotes

^1^http://www.phylogeny.fr/

^2^http://www.brain-map.org
